# Varroa Appears to Drive Persistent Increases in New Zealand Colony Losses

**DOI:** 10.3390/insects13070589

**Published:** 2022-06-28

**Authors:** Philip Stahlmann-Brown, Richard J. Hall, Hayley Pragert, Thomas Robertson

**Affiliations:** 1 Manaaki Whenua, Landcare Research, Wellington 6011, New Zealand; trobe745@gmail.com; 2 Biosecurity New Zealand, Ministry for Primary Industries, Wellington 6011, New Zealand; richard.hall@mpi.govt.nz (R.J.H.); hayley.pragert@mpi.govt.nz (H.P.)

**Keywords:** honey bee, colony loss, *Apis mellifera*, varroa

## Abstract

**Simple Summary:**

New Zealand is a significant exporter of high-value honey, and honey bees are the major pollinator of many important food crops. Bee colonies naturally die over winter due to the pressures of the season, and we have been surveying beekeepers annually since 2015 to record these losses. The percentage of colonies that died over winter increased every year between 2015 and 2021. While problems with queen bees were previously the main issue to which beekeepers attributed losses, 2021 was the first year in which beekeepers identified the parasitic varroa mite as the main cause. The mite invaded New Zealand in 2000; despite being in the country for more than 20 years, New Zealand beekeepers are still struggling to control varroa.

**Abstract:**

New Zealand’s temperate climate and bountiful flora are well suited to managed honey bees, and its geographic isolation and strict biosecurity laws have made sure that some pests and diseases affecting bees elsewhere are not present. Nevertheless, given the importance of pollination and high-value export honey to the economy, New Zealand began systematically measuring winter colony losses in 2015. The New Zealand Colony Loss Survey is modelled on the COLOSS survey but has been adapted to the New Zealand apicultural context. Some 49% of New Zealand beekeepers completed the winter 2021 survey. Between 2015 and 2021, overall colony loss rates increased monotonically from 8.37% [95% CI: 7.66%, 9.15%] to 13.59% [95% CI: 13.21%, 13.99%]. Whereas beekeepers most commonly attributed losses to queen problems between 2015 and 2020, attributions to varroa have escalated year-on-year to become the largest attributed cause of colony loss. Losses to varroa are perhaps amplified by the 23.4% of respondents who did not monitor mite loads and the 4.4% of beekeepers who did not treat varroa during the 2020/21 season. Indeed, most beekeepers consider their treatment to be effective and note that treating at the wrong time and reinvasion were major drivers of losses to varroa.

## 1. Introduction

The pernicious effects of honey bee pests and diseases have compelled many countries to undertake annual surveys of colony losses for managed honey bees, *Apis mellifera* [1]. These surveys first started in North America in 2007 in response to reports that varroa was becoming resistant to treatments. The Canadian Association of Professional Apiculturists has performed its survey every year since 2007 [2]. Dramatic and sudden reports of substantial winter colony losses in the USA in 2006—rates in excess of 35%—also compelled the need for an annual survey of winter colony losses. The USA colony loss survey has been conducted every year since 2007, the effort being sustained by the Bee Informed Partnership [3,4]. Europe, the Middle East, Africa, and Asia have all also reported high levels of overwintering colony losses, and countries in these regions initiated annual surveys of colony losses [5,6,7,8,9] under the auspices of COLOSS (Prevention of honey bee COlony LOSSes) [10]. COLOSS established a working group in 2008 that developed a standardized survey to ensure that colony loss data collected by different member states is comparable [6].

Given the important role that honey bees play in New Zealand agricultural, horticultural, and export industries, an annual survey of winter colony losses was commissioned by the New Zealand Ministry for Primary Industries in 2015. The New Zealand Colony Loss Survey is modelled on the international COLOSS survey, but with bespoke design modifications that also accommodate issues specific to the New Zealand apicultural context.

New Zealand spans 12 degree of latitude and has a temperate climate, experiencing only a mild winter. The country has a diversity of native and exotic flora that provide abundant pollen and nectar resources, allowing honey bee colonies to flourish at comparatively high stocking rates [11]. Of particular note, native trees in the *Myrtaceae* family (e.g., *Leptospermum scoparium* (mānuka), *Metrosideros excelsa* (pōhutukawa), *Metrosideros robusta* (rātā), and *Kunzea ericoides* (kānuka)) provide honey bees with substantial seasonal nectar yields and provide monofloral honey crops. Exotic flora also make a substantial contribution to honey crops or pollen sources, with examples such as *Trifolium repens* (clover), eucalyptus, willows, *Chamaecytisus palmensis* (tree lucerne), and *Ulex europaeus* (gorse).

**Figure 1:**
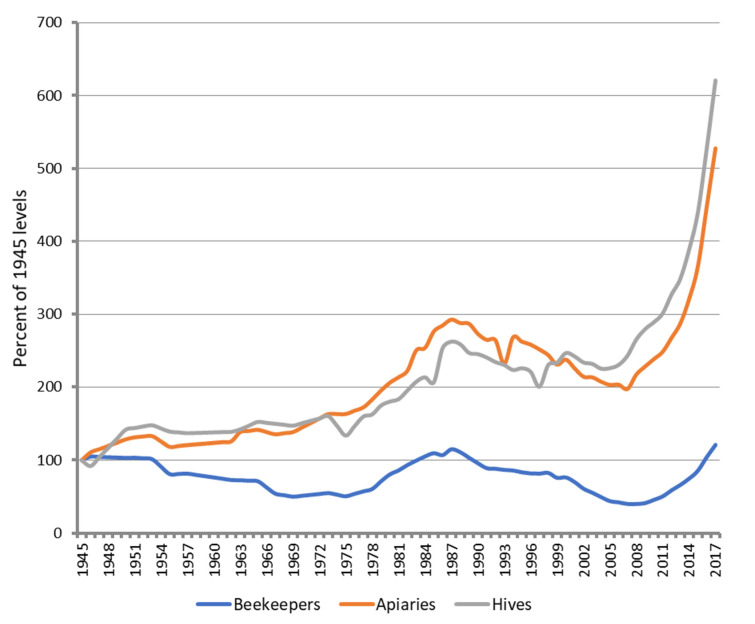
The number of beekeepers, apiaries, and colonies in New Zealand from 1945–2017. Values on the *y*-axis are expressed as a percentage of the number of colonies reported in the year 1945 (Source, AsureQuality, New Zealand).

While honey bees were introduced to New Zealand in 1839, hive numbers in New Zealand have been systematically recorded since 1945, and both the number of apiaries and the number of colonies have increased exponentially since 2006 (Figure 1). Economic reward underlies the burgeoning of the honey bee population: mānuka honey, with its documented antimicrobial effects [12,13,14], commands significant price premiums and provides a substantial export for New Zealand, with a record export of 12,788 tonnes worth NZ$482 million (At the time of writing, NZ$1 = €0.63) in 2021 [15]. Bees also provide critical pollination services to an expanding horticultural industry that produces apples, pears, apricots, peaches, kiwifruit, avocados, carrots, onions, and other food crops. Managed honey bees provide at least NZ$5 billion worth of pollination services annually in New Zealand [16]. There were 806,140 colonies in New Zealand as of 1 June 2021 [17], a ratio of approximately one hive to every six New Zealand residents.

The number of beekeepers in New Zealand decreased from the year 1945 through to the mid 1970s, when beekeeping enjoyed a brief resurgence, falling again from the late 1980s through to 2008. Since 2008, the number of registered beekeepers in New Zealand has increased monotonically—albeit at a much lower rate of increase than either colonies or apiaries—spurred by a renewed interest in recreational beekeeping. There were 9891 registered beekeepers with at least one colony as of June 2021 [17].

The number of active commercial beekeepers (as of June 2021, 530 beekeepers reported having registered beekeeping operation sizes greater than 250 colonies) and the concentration of colonies under the management of a relatively small number of operators (as of June 2021, 5.7% of beekeepers operated 84.8% of New Zealand’s production colonies) distinguish New Zealand apiculture [17]. For example, eleven commercial operators in New Zealand have more than 10,000 registered colonies. In contrast, less than 0.1% of beekeepers in Germany had more than 150 colonies and just 50 out of 37,888 beekeepers in the UK had more than 150 colonies as of 2017 per the European Parliamentary Research Service [18]. As of 2013, 6% of beekeepers in the European Union had more than 150 colonies and 2% had more than 300 colonies [19]. In Canada, 20% of beekeepers maintain 80% of colonies according to a 2022 report on the Canadian apiculture industry [20].

Situated over 4000 km to the east of Australia, New Zealand is an isolated island nation. Its strict biosecurity laws do not allow for the importation of live bees or the import of bee products, as these pathways may expose the national hive stock to biosecurity risks such as European foulbrood (*Melissococcus plutonius*), small hive beetle (*Aethina tumida*), Tropilaelaps mites (*Tropilaelaps clareae* and *T. mercedesae*), tracheal mite (*Acarapis woodi*), and Israeli acute paralysis virus [21]. Lacking a mechanism for restocking bees via importation should colony loss rates become very high, New Zealand began systematically monitoring annual winter colony losses in 2015.

In this paper, we review trends and observations of winter colony losses in New Zealand from 2015 through to 2021. Specifically, we document the emergence of the parasitic mite *Varroa destructor* as an increasingly frequently identified driver of New Zealand’s rising colony loss rates.

## 2. Materials & Methods

### 2.1. Survey Design

The New Zealand Colony Loss survey has been conducted annually since 2015. Adapted from the standard survey questionnaire administered by COLOSS, the 2015 survey [22] focused on overwinter colony losses. It also collected information on queen performance, indicators of pests and diseases, varroa treatment, and colony management. Because the challenges facing beekeepers in temperate, geographically isolated New Zealand differ from those facing beekeepers in Europe and elsewhere in the northern hemisphere, the questionnaire was amended to include questions relevant to regional concerns. Questions about the methods used to monitor for varroa were added in 2016 [23]. 

The 2017 questionnaire [24] included two important refinements. First, COLOSS surveys include a category of losses entitled “dead colonies or empty hives”, which explicitly includes “suspected toxic exposure” and “suspected starvation” and implicitly includes both varroa and diseases [25]. New Zealand beekeepers considered this categorization to be confusing and requested greater clarity in subsequent surveys; hence, beginning in 2017, beekeepers were asked to attribute losses to specific causes (e.g., starvation and exposure to toxins) without first asking beekeepers to report “dead colonies”. In addition, we allowed for other important explanations for colony loss including “suspected varroa and related complications”, “suspected nosema and other diseases”, and “robbing by other bees”. These categories overlap significantly with those included in other colony loss surveys, e.g., that undertaken by the Bee Informed Partnership in the US [26]. Regardless, we acknowledge that these are beekeepers’ own attributions of losses rather than the results of laboratory testing.

In 2020 [27], the recording of winter losses was simplified by focusing on four high-level categories: unresolvable queen problems, natural disasters and accidents, theft or vandalism, and colonies that were dead upon inspection. Beekeepers who indicated that they had colonies that were dead upon inspection were then asked to specify the nature of those deaths. This re-framing of the questionnaire aligns more closely with the COLOSS questionnaire but also resolves local beekeepers’ difficulties with question wording.

The 2021, New Zealand Colony Loss Survey [28] focused more acutely on varroa. Specifically, respondents were asked to describe monitoring and treatment in considerable detail. They were also asked to report why varroa had caused overwinter losses.

### 2.2. Survey Enumeration

The New Zealand Colony Loss Survey is administered to beekeepers via the Qualtrics survey platform. Electronic survey enumeration affords several advantages over alternative data-collection methods: in particular, it reduces respondent burden via branching and ensuring the relevance of each question to each respondent [29]. For example, only those beekeepers who lost production colonies over winter were asked to provide details on the nature of those losses. In addition, electronic enumeration eliminates data-entry error, increasing the accuracy of results for analysis [30].

One criticism of online surveys is that they may compromise accessibility, particularly for rural populations, including beekeepers. However, it is projected that 99.8% of New Zealand’s population will have broadband Internet access by the end of 2022 [31]. Moreover, the survey was optimized for portable devices such as tablets and phones to increase accessibility for those without high-speed Internet access at home. The survey was also made available via telephone interview to reach beekeepers who lack Internet access.

### 2.3. Survey Sample

Under the Biosecurity Act of 1993, all New Zealand beekeepers are legally required to register their apiaries and to complete an Annual Disease Return by 1 June each year. Virtually all beekeepers include email addresses in their registrations, and hence personalized invitations to participate in the survey are sent to all New Zealand beekeepers for whom email addresses were available. Reminders are sent semi-monthly to any beekeeper who has not yet completed the survey. In addition, we encourage large beekeeping operations to participate in the survey via personalized telephone calls, beginning in October, approximately five weeks after the survey opens. Third, we offer prize draws for grocery vouchers to encourage participation.

New Zealand has one of the highest response rates of colony loss surveys worldwide; since 2016, between 30.9% and 49.1% of all registered beekeepers completed the survey in any given year. Overall, participating beekeepers manage between 30.1% and 47.2% of all registered colonies (Table 1).

### 2.4. Estimating Colony Losses and Confidence Intervals

Per Van der Zee et al. [32], there are two standard approaches to calculating loss rates. The “overall loss rate” is measured as winter losses summed across beekeepers divided by the number of colonies that were alive at the beginning of winter, also summed across beekeepers. The “average loss rate” is the average of each respondent’s winter losses divided by their living colonies at the beginning of winter. The latter approach weighs losses equally across beekeepers even though loss rates vary across operation size. Thus, van der Zee et al. [32] recommends reporting overall loss rates rather than average loss rates, and thus COLOSS [8,33], the Bee Informed Partnership [3,4], and the New Zealand Colony Loss Survey [28] all report overall loss rates.

Confidence intervals are often calculated using a binomial distribution. However, this approach implies that the likelihood of survival for any given colony is independent of that for any other colony and that the probability of survival is the same for all colonies, ignoring the fact that the performance of any one colony in an apiary depends on the performance of other colonies in the same apiary [34]. Such clustering of losses can lead to under or over dispersion in the data [34], which can affect standard errors and confidence intervals [8]. Thus, we follow our European [8,33] and American [3,4] colleagues in reporting standard errors based on a quasi-binomial distribution and a logit link function, which derives a confidence interval for the overall loss rate based on the standard error of the estimated intercept in a model with only an intercept [32,34,35]. 

## 3. Results

### 3.1. Colony Losses in New Zealand from 2016–2021

Overall colony loss rates have increased during the seven-year study period, rising from 8.37% [95% CI: 7.66%, 9.15%] in 2015 to 13.59% [95% CI: 13.21%, 13.99%] in 2021 (Table 2). While colony loss rates have fluctuated year-on-year within regions, an upward trend in the colony loss rate is generally observed for both the North Island and South Island.

### 3.2. Beekeeping Operation Size and Colony Loss Levels

**Figure 2:**
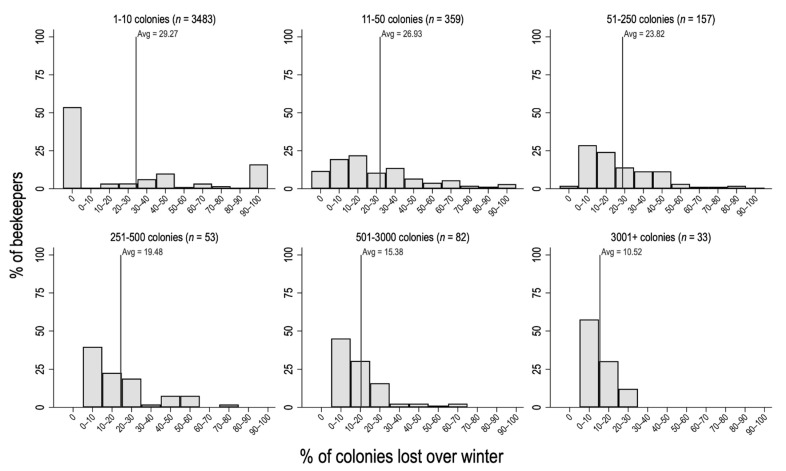
Distribution of average colony losses over winter, by operation size. The *x*-axis shows the percentage of colonies lost, and the *y*-axis shows the percentage of beekeepers within each class. Six operation size categories were selected for analysis, those being hobbyist beekeepers with 1–10 colonies, smaller operation sizes of 11–50 colonies and 51–250 colonies, small commercial operations of 251–500 colonies, medium operation sizes of 501–3000 colonies, and large operation sizes of more than 3000 colonies.

Respondents to the survey represent a cross-section of the New Zealand beekeeping industry. For example, in 2021, 3483 respondents had fewer than ten colonies going into winter compared to 684 respondents with larger operations (Figure 2). Hobbyist beekeepers with fewer than ten colonies report higher average colony loss rates, but the distribution is bimodal in which a large proportion (>50%) lost no colonies during the winter but >10% lost all of their colonies over winter. Colony loss rates in larger operations are more evenly distributed, with losses of less than 10% being commonly observed (Figure 2).

### 3.3. Factors Attributed to the Loss of Colonies during Winter

The most common cause that beekeepers attribute to winter colony losses was previously queen problems (e.g., poorly mated queens, old queens), which accounted for between 30.3% and 35.7% of attributions of losses between 2015 and 2020 (Table 3). However, losses attributed to varroa infestation have escalated year-on-year, beginning at 16.9% in 2017 and surpassing queen problems to become the largest attributed cause of colony loss at 38.9% in 2021. Suspected starvation and the effects of wasps are cited as the other two substantial causes, with starvation showing a downward trend in attribution. Other attributions of losses (natural disasters, vandalism, AFB, Argentine ants, robbing by other bees, toxin exposure, nosema and other disease) are significantly less common and relatively stable over time; hence, they have been aggregated together in Table 3.

### 3.4. Varroa Mite Monitoring 

**Figure 3:**
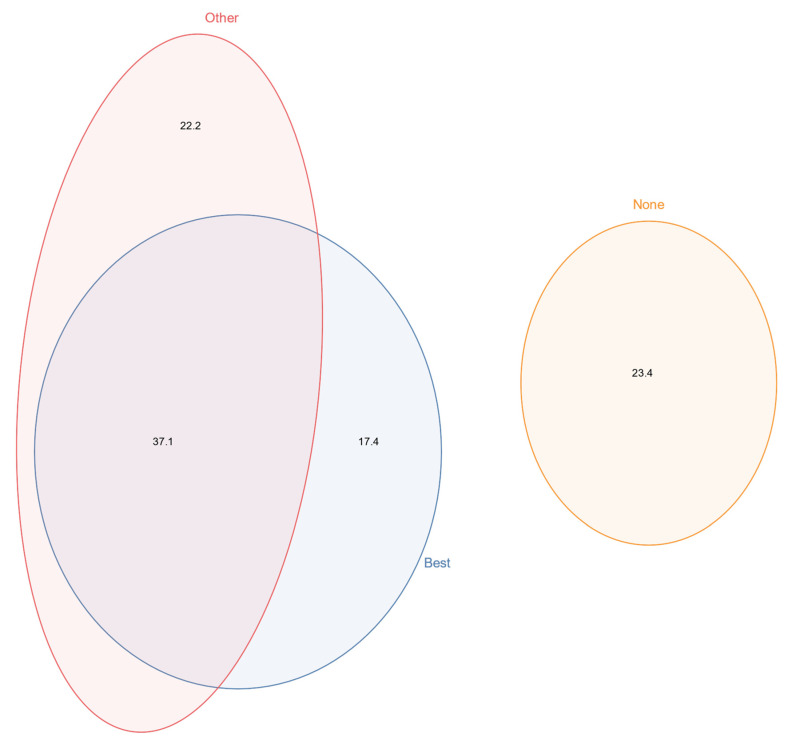
Euler plot showing the percentage of the three categories of varroa monitoring methods that beekeepers reported to have used in winter 2021. Best: alcohol wash, sugar shake, CO_2_ injection, and sending samples to a lab. Other: includes any other method. None: defined as no monitoring, or if a beekeeper cited “visual inspection of adult bees” as a method for monitoring.

Most New Zealand beekeepers did monitor for varroa during the 2020/21 season. 57.3% of the beekeepers indicated that they used any of the internationally recognized and accurate methods for assessing varroa infestation (Figure 3), those being alcohol/soap wash, sugar shake, CO_2_ injection, and sending samples to a lab [36,37,38,39,40], sometimes in combination with less preferred monitoring methods such as sticky boards and visual inspection of drone brood. Some 17.4% of respondents undertook one of these two monitoring methods but not one of the preferred methods. Beekeepers who report relying exclusively on visual inspection of adult bees for monitoring varroa are grouped with those who report undertaking no monitoring for varroa; this group comprised 23.4% of the sample in 2021.

### 3.5. Varroa Treatment in 2021

**Figure 4:**
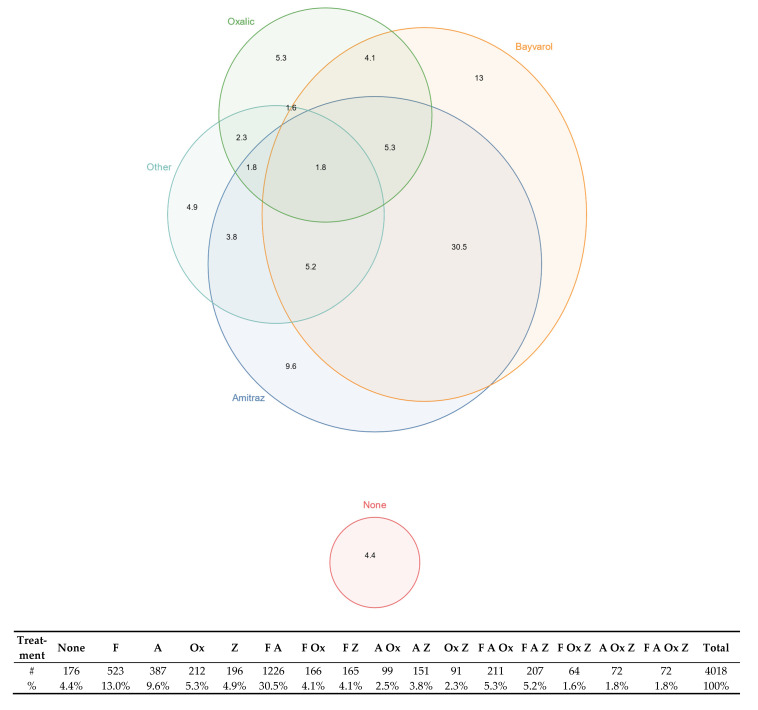
Euler plot showing the percentage of beekeepers using specific treatments between spring 2020 and winter 2021 alongside tabulated figures in the table that show the percentage of beekeepers within each category. F: flumethrin. A: amitraz. Ox: oxalic acid. Z: other treatments.

Varroacides can substantially reduce colony loss rates, but treating varroa is costly for beekeepers in terms of both labor and materials. For example, synthetic varroacides purchased in bulk in New Zealand cost between NZ$8.00 and NZ$11.25 per colony treated. Installation and removal of the strips requires at least two visits to an apiary that may or may not otherwise have been needed. Some 4.4% of beekeepers reported that they did not treat for varroa at all during the 2020/21 season (Figure 4).

For those beekeepers who do treat for varroa, flumethrin (marketed in New Zealand as Bayvarol^®^), amitraz (marketed as Apivar^®^ and Apitraz^®^), and oxalic acid (whether in the form of sublimation/vaporisation, dribbling/trickling, or glycerine strips/staples) are by far the most common forms of varroa treatment in New Zealand. Between spring 2020 and winter 2021, 13.0% of beekeepers treated with flumethrin only, 9.6% treated with amitraz only, 5.3% used oxalic treatments only, and 4.9% relied exclusively on other treatment types. For beekeepers who used more than one type of treatment, the most common combination was amitraz and flumethrin, with 30.5% of beekeepers reporting this combination. 5.3% of beekeepers reported treating with flumethrin, amitraz, and oxalic acid, and 1.8% reported using flumethrin, amitraz, oxalic acid, and other treatments.

**Figure 5:**
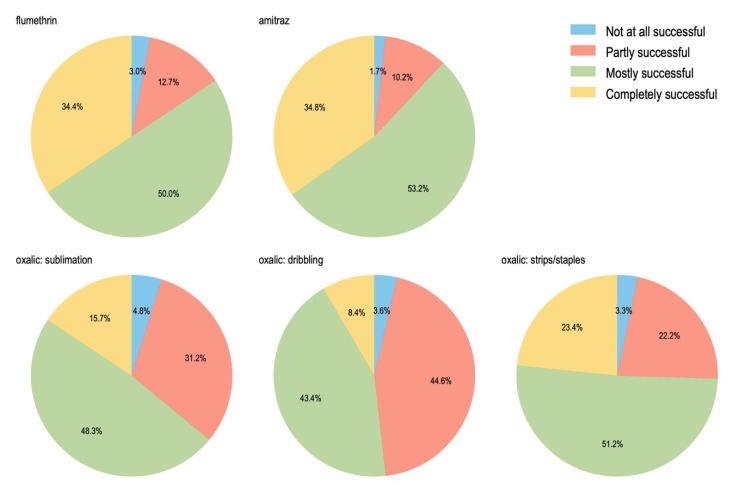
Beekeepers’ self-reported effectiveness of varroacides in managing varroa infestation, rated as having been completely successful, mostly successful, partly successful or not at all successful during the winter of 2021. The most common varroacide treatments in New Zealand are flumethrin, amitraz, and oxalic acid. Oxalic acid was divided into sub-categories, based on the three most common methods of application: sublimation, dribbling, or the in-hive placement of strips/staples that have been soaked in oxalic acid.

When asked about the successfulness of the three most common types of varroacides (i.e., flumethrin, amitraz and oxalic acid), the majority of beekeepers indicated that treatments were either completely successful or mostly successful (Figure 5). The synthetic varroacides were generally reported as being more successful than oxalic acid, with a substantial proportion of those respondents who used oxalic acid reporting that treatment was only partially successful.

**Figure 6:**
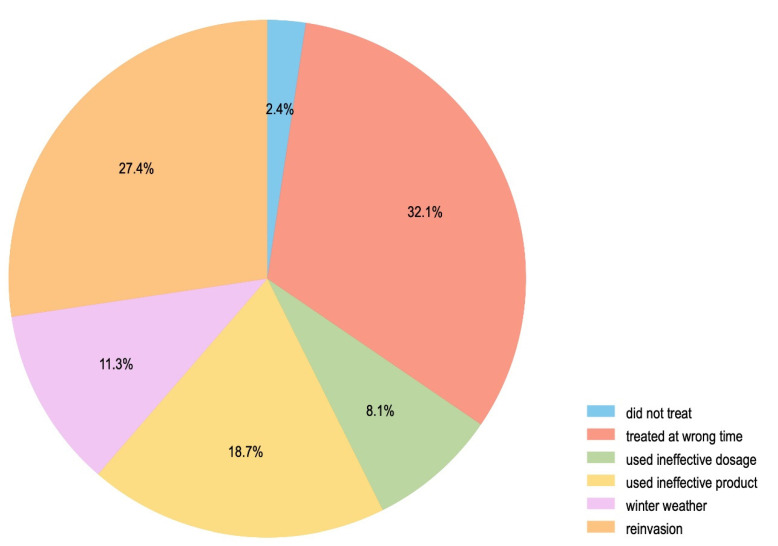
Reasons given for colony losses attributed to varroa during winter 2021 .

Finally, beekeepers who attributed overwinter losses to suspected varroa were asked to identify the primary reason. Most beekeepers cited reinvasion or treating at the wrong time as the reason for varroa treatment failures they had observed (59.5% combined, Figure 6). A lesser proportion (18.7%) said they had used an ineffective product. A smaller group of respondents reported either that they experienced bad weather (11.3%) or that they had used an ineffective dose of a treatment (8.1%; Figure 6).

### 3.6. Loss Rates That Beekeepers Consider Acceptable

**Figure 7:**
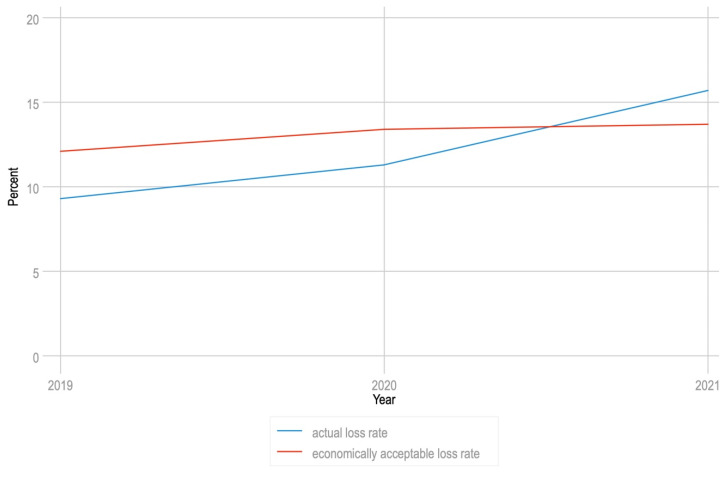
Trend in acceptable colony loss rate in comparison to reported colony loss rates between 2019 and 2021, for beekeepers with >250 colonies.

Beekeepers with more than 250 colonies were asked to nominate a winter colony loss rate that they considered to be acceptable from an economic perspective (Figure 7). Beginning in 2021, the realized colony loss rate surpassed what beekeepers deem to be economically acceptable (Figure 7), suggesting that New Zealand beekeepers’ capacity to absorb rising loss rates is waning.

## 4. Discussion

Over-winter colony loss rates have risen monotonically in New Zealand since formal surveying began in 2015 [28]. Although queen performance has been cited as a prominent reason for colony loss since the inception of the New Zealand Colony Loss Survey [22,23,24,27,28,41,42], it is the deleterious effect of varroa that is causing increasing concern. Indeed in 2021, attributions of losses to varroa exceeded attributions to queen problems for the first time, and the trend is pointing upwards despite beekeepers having had 22 years’ experience in dealing with the mite since its arrival in New Zealand.

New Zealand thus joins other countries in which varroa is a major factor underlying colony losses, together with queen problems and starvation. For example, more than 60% of commercial and semi-commercial beekeepers responding to a US survey reporting that varroa was a cause of colony loss during winter 2020/2021 [26]. In addition, approximately 50% of commercial beekeepers and more than 40% of semi-commercial beekeepers reported that queen problems contributed to their losses, while nearly 20% of commercial beekeepers and more than 30% of semi-commercial beekeepers reported that starvation contributed to their losses.

Low levels of monitoring potentially contribute to New Zealand’s increasing losses to varroa. For example, overwinter losses were estimated to be 14.7% among Austrian beekeepers who monitored for varroa prior to winter 2018/19, compared to 21.7% for those who did not monitor [43]. Indeed, nearly one-quarter of all New Zealand beekeepers forwent formal monitoring during the 2020/21 season.

Resistance to miticides is often cited as an important factor in colony losses attributed to varroa [2,44], and such resistance has been widely observed in both North America and Europe [45,46,47,48]. In contrast, most New Zealand beekeepers observed that the timing of treatment or reinvasion of varroa from neighboring apiaries had caused the varroa-attributed losses, not resistance. In fact, most beekeepers considered their treatment to be “mostly successful” or “completely successful”.

Greater adoption of best practices for monitoring could clearly help beekeepers to resolve problems with the timing of their varroa treatments. Reinvasion is likely more difficult to address given the finding that 4.4% of New Zealand beekeepers are not treating for varroa. It is difficult to mitigate reinvasion if the surrounding apiary landscape has high levels of varroa infestation because mite levels can quickly re-establish to economically damaging levels after treatment [49]. Reinvasion, the timing of treatment, the presence of non-treatment apiaries, and the lack of effective mite monitoring, when considered together, are likely to form an antagonistic quadrat that sustains the persistent pressure of varroa on New Zealand honey bee colonies.

Within New Zealand, efforts to raise awareness about the effects of varroa, varroa monitoring, and varroa treatment are ongoing. The parasite is a central topic of discussion at national apiculture conferences, within apiculture publications [50], and by industry groups. The New Zealand Colony Loss Survey has also drawn attention to the challenges posed by varroa [51]. New Zealand beekeepers continue to work on breeding for varroa-sensitive hygiene (https://www.beesmartbreeding.co.nz/sales-services (accessed on 18 May 2022), https://www.bettabees.co.nz/research (accessed on 18 May 2022)), and a variety of extension materials (https://www.mpi.govt.nz/biosecurity/how-to-find-report-and-prevent-pests-and-diseases/bee-biosecurity/ (accessed on 18 May 2022), https://www.mpi.govt.nz/dmsdocument/47824-The-New-Zealand-Honey-Bee-Longitudinal-Study (accessed on 18 May 2022)) are available for beekeepers that describe best-practice methods for monitoring and treating varroa, including an updated varroa manual [52]. Varroa also features as an important part of apicultural training programmes within New Zealand, and research into the dynamics of transmission and effects of the parasite are ongoing [53,54].

The economic drivers of beekeeping in New Zealand are somewhat unique in that honey—not pollination—is the primary source of income derived from beekeeping. Cost pressures have been increasing for New Zealand beekeepers, with the value of some non-mānuka honeys now at or below the cost of production (NZ$3–NZ$7 per kg), due at least in part to substantial honey harvests in previous years and significant honey reserves [15]. Even though the total honey export volume reached record highs in 2021, the average price per kg fell by 9% compared to the previous year [15], and approximately 7% of commercial beekeeping enterprises exited the industry in 2021 [17]. Therefore, it is possible that some beekeepers sought to cut upfront operational costs by reducing or eliminating varroa treatment.

Varroa is likely to persist as an insidious threat to *Apis mellifera* in New Zealand. Moreover, as a vector of Deformed Wing Virus and other viral pathogens [55] to wild bees—of which New Zealand has at least 27 endemic species [56]—varroa also poses a significant threat to bee biodiversity in our island ecosystem. Effective varroa control is required for maintaining the integrity of New Zealand’s apiculture industry and for mitigating mounting disease pressures in bee populations, both wild and managed.

All of these results notwithstanding, it is important to note that our survey data were not verified in the field or by using laboratory testing methods. We thus advocate for assessing the factors to which beekeepers attribute their colony losses alongside studies that employ field inspections or diagnostic testing [21,57,58,59].

## Figures and Tables

**Table 1:** New Zealand Colony Loss Survey Response Rates, between the Years 2015–2021.

Year	# of Respondents	% of All Registered Beekeepers	# of Colonies Reported	% of All Registered Colonies
2021	4355	49.10%	381,148	47.20%
2020	2863	32.00%	304,143	34.70%
2019	3456	36.70%	297,377	36.20%
2018	3655	42.30%	365,986	41.60%
2017	2066	30.90%	242,926	30.10%
2016	2179	37.90%	275,356	40.30%
2015	366	6.70%	225,660	39.60%

**Table 2:** Overall colony loss rates reported at the national-level in the New Zealand Colony Loss Survey between the years 2015 and 2021.

Year	Overall Loss Rate	95% CI
2021	13.59%	[13.21%, 13.99%]
2020	11.30%	[10.95%, 11.66%]
2019	10.41%	[10.05%, 10.77%]
2018	10.20%	[9.85%, 10.57%]
2017	9.70%	[9.37%, 10.05%]
2016	9.53%	[9.07%, 10.02%]
2015	8.37%	[7.66%, 9.15%]

**Table 3:** Losses attributed to queen problems, varroa, and other factors, between the years 2015–2021 *.

Year	Queen Problems	Suspected Varroa	Suspected Starvation	Wasps	All Other Causes
2021	24.0%	38.9%	7.0%	12.0%	18.1%
2020	33.1%	31.0%	7.6%	6.6%	21.7%
2019	30.3%	28.1%	10.3%	9.6%	21.7%
2018	35.5%	19.5%	12.1%	12.1%	20.8%
2017	34.4%	16.9%	13.9%	9.7%	25.1%
2016	29.3%	see note *	17.2%	11.7%	41.8%
2015	35.7%	see note *	15.6%	14.9%	33.8%

* The questionnaire was substantially revised in 2017. Therefore, the attributions of losses for 2015 and 2016 may not be directly comparable to those for 2017 onwards. Suspected varroa was included in the category “all other causes” for the 2016 and 2017 survey.

## Data Availability

The data presented in this study are available on request from the corresponding author. The data are not publicly available due to privacy concerns and potential commercial sensitivities.
